# Adjuvant-dependent protection of SARS-CoV-2 spike vaccines: comparative immunogenicity of human-applicable formulations

**DOI:** 10.1128/jvi.01099-25

**Published:** 2025-10-03

**Authors:** Zhendong Pan, Liangliang Jiang, Yingying Chen, Haoran Peng, Yangang Liu, Xu Zheng, Yanhua He, Yan Liu, Ying Wang, Xiaoyan Zhang, Zhongtian Qi, Cuiling Ding, Jianqing Xu, Ping Zhao

**Affiliations:** 1 Department of Microbiology, Faculty of Naval Medicine, Naval Medical University 12521, Shanghai, China; 2 Department of Microbiology and Immunology, Shanghai Institute of Immunology, Shanghai Jiao Tong University School of Medicine 56694, Shanghai, China; 3 Shanghai Institute of Virology, Shanghai Jiao Tong University School of Medicine 56694, Shanghai, China; 4 Shanghai Public Health Clinical Center & Institutes of Biomedical Sciences, Fudan University 12478, Shanghai, China; Loyola University Chicago - Health Sciences Campus, Maywood, Illinois, USA

**Keywords:** SARS-CoV-2, spike protein, adjuvant, cross-protection, booster immunization, immunosenescence

## Abstract

Although recombinant vaccines with various adjuvant systems are widely deployed in global coronavirus disease 2019 immunization, their distinct immune profiles have not been fully elucidated. In this study, we evaluated immune responses induced by four clinically validated adjuvants—aluminum hydroxide (Al), two water-in-oil emulsions (Montanide ISA720 and ISA51), and an oil-in-water emulsion (Sepivac SWE)—formulated with the prefusion-stabilized ancestral severe acute respiratory syndrome coronavirus 2 (SARS-CoV-2) spike trimer (S-2P) in mouse models. Both S-2P:ISA720 and S-2P:ISA51 elicited potent humoral immunity, including cross-neutralizing antibodies against Omicron variants, along with robust interferon gamma-producing T-cell responses in the spleen and lung. Immunization with S-2P:ISA720 conferred complete protection against lethal challenge with the ancestral virus as well as Omicron subvariants BA.5 and BF.7. In contrast, S-2P:Al and S-2P:SWE elicited substantially weaker antibody responses, undetectable T-cell immunity, and markedly reduced protective efficacy. Prime-boost immunization with S-2P:ISA720 induced sustained peak antibody titers against both homologous SARS-CoV-2 and the Omicron BA.2 subvariant, affording complete protection for up to 17 weeks post-vaccination. While a third dose of S-2P:Al following a prime-boost regimen triggered a robust increase in antibody levels, titers declined rapidly thereafter, recapitulating the decay kinetics observed after the second dose. Both ISA720 and Al formulations exhibited age-dependent declines in antibody responses and protective efficacy. Notably, aged mice displayed markedly attenuated neuroinflammatory responses following SARS-CoV-2 challenge and significantly compromised protection after adoptive transfer of immune serum. These results underscore adjuvant-specific determinants of broad and durable immunity and reveal novel age-related constraints in vaccine-induced protection against SARS-CoV-2.

**IMPORTANCE:**

Persistent viral evolution, rapid waning of vaccine-induced immunity, and the heightened vulnerability of elderly populations remain major challenges for COVID-19 vaccination strategies. In this study, we systematically assessed immune responses elicited by the ancestral spike protein formulated with four distinct adjuvants in mouse models. We demonstrate that an optimized adjuvant formulation markedly enhances the magnitude and breadth of antibody responses, potentiates T-cell immunity, and rapidly induces sustained peak antibody titers against both homologous virus and Omicron variants. Vaccine-induced antibody responses were significantly attenuated in aged mice, and furthermore, both the protective efficacy of antibodies and inflammatory cytokine responses upon viral challenge were impaired in aged animals. These results provide compelling evidence that rational adjuvant selection is critical for enabling recombinant vaccines to achieve rapid-onset, broad, and durable immune protection. Furthermore, our study offers new mechanistic insights into the reduced vaccine efficacy observed in the elderly.

## INTRODUCTION

The coronavirus disease 2019 (COVID-19) pandemic, caused by severe acute respiratory syndrome coronavirus 2 (SARS-CoV-2), has led to great damage to human health and the world’s global social and economic development and still remains a threat to public health worldwide. Since the beginning of 2025, a resurgence of SARS-CoV-2 infections has occurred in numerous locations worldwide. Vaccination is the most effective strategy for controlling infectious diseases. The SARS-CoV-2 spike protein enables viral cell entry by binding its S1 subunit’s receptor-binding domain (RBD) to the human cellular receptor angiotensin-converting enzyme 2 (ACE2) (1, 2). Thus, the spike protein and its RBD function as principal targets for neutralizing antibodies and serve as critical vaccine antigens (3–5). Several vaccines were rapidly developed and approved for mass vaccination worldwide. These vaccines have shown efficacy against SARS-CoV-2 infection, particularly in reducing severe disease, hospitalizations, and mortality (6–10). However, immune-evading variants that largely occurred in the viral spike protein continue to emerge. Notably, since late 2021, Omicron and its subvariants have shown pronounced antibody evasion, posing a significant challenge to worldwide vaccination campaigns (11–13). Furthermore, the neutralizing antibody elicited by natural infection and vaccination generally rapidly decreases over time (14–16). Older people are particularly susceptible to SARS-CoV-2 infection and develop severe disease due to the impaired adaptive immunity to vaccination, dysregulated inflammatory response, and age-related chronic diseases (17–19). Multiple strategies have been adopted to combat the challenges of rapidly evolving viral variants and waning immune protection, including booster vaccinations, antigen updates in vaccine formulations, and the development of multivalent antigen-based vaccines (13, 20–22).

Currently, three distinct vaccine platform technologies—inactivated virus vaccine (6), mRNA-based lipid nanoparticle vaccine (7, 8), replication-incompetent adenovirus vector vaccine (9), and adjuvanted recombinant protein vaccines (10)—are commercially available for COVID-19 prevention. Among these, adjuvanted recombinant protein vaccines exhibit unique advantages, including superior safety profiles, enhanced stability, greater cost-effectiveness, and improved manufacturing scalability. These benefits position recombinant protein vaccines as a particularly valuable option for global immunization efforts.

Adjuvants serve as crucial components of recombinant vaccines, orchestrating both antigen presentation to immune cells and delivery of immunostimulatory signals required for effective immune priming (23). Adjuvants profoundly influence the magnitude, breadth, and quality of immune responses to vaccine antigens. Although diverse adjuvant systems, from conventional aluminum salts to newer formulations like the oil-in-water (O/W) emulsion AS03 and saponin-based Matrix-M nanoparticles, have been employed in recombinant COVID-19 vaccines (10, 24–26), the respective unique immune characteristics induced by these distinct adjuvants remain to be fully elucidated.

Aluminum salt adjuvants (principally aluminum hydroxide and aluminum phosphate) are the most widely used adjuvants in human vaccines. They function through multiple mechanisms, including the formation of an antigen depot enabling sustained release, the induction of local inflammatory responses, and the activation of innate immune pathways (23, 27). While highly effective at enhancing antibody-mediated immunity, these adjuvants predominantly elicit Th2-skewed immune responses with weak cellular immunity induction. This Th2 bias raises potential safety considerations for respiratory virus vaccines, as it may potentially contribute to vaccine-associated enhanced respiratory disease (VAERD) in certain cases (28).

Water-in-oil (W/O) emulsion adjuvants demonstrate superior immunogenicity compared to aluminum salt adjuvants, particularly in eliciting T-cell-mediated immunity, and their application in human vaccines has been steadily increasing (23, 29). Among these, Seppic’s Montanide ISA 720 and ISA 51 represent well-established W/O emulsion platforms that have undergone extensive clinical evaluation over several decades for various vaccine candidates against infectious diseases and cancers (29, 30). These adjuvants have proven effective in enhancing both antigen-specific antibody production and cytotoxic T-lymphocyte responses. The immunostimulatory properties of these adjuvants primarily involve antigen depot formation, localized inflammatory responses, and lymphocyte trapping at the injection site. While both are W/O emulsions, they differ substantially in composition. ISA 720 employs metabolizable squalene as its oil phase, offering excellent biocompatibility, whereas ISA 51 uses non-metabolizable mineral oil that typically provokes stronger local inflammation, a characteristic that may contribute to its enhanced capacity to stimulate T-cell-mediated immunity. Sepivac SWE represents a next-generation O/W emulsion platform that is similar to another O/W adjuvant MF59 (31). All three adjuvants (ISA 720, ISA 51, and SWE) have demonstrated favorable safety profiles and immunogenicity in I/II clinical trials of SARS-CoV-2 vaccine candidates (32–35).

In this study, we evaluated aluminum hydroxide and Seppic’s Montanide ISA 720, ISA 51, and Sepivac SWE—combined with the prefusion-stabilized ancestral SARS-CoV-2 spike protein trimer (S-2P) in wild-type C57BL/6 mice and congenic humanized ACE2 transgenic mice. Our assessment included multiple protective immunity parameters: (i) magnitude and breadth of immune responses, (ii) durability of immunity, (iii) protective efficacy in aged mice, and (iv) effects of booster immunization.

## MATERIALS AND METHODS

### Cells, viruses, and proteins

HEK293T and HEK293 cells were obtained from the American Type Culture Collection (ATCC). The HEK293 cells stably expressing human ACE2 (HEK293-ACE2) were generated by infecting HEK293 cells with a human ACE2 lentivirus, followed by puromycin selection. Vero E6 cells were kindly provided by Dr. Rong Zhang of Fudan University. HEK293T, HEK293-ACE2, and Vero E6 cells were cultured in Dulbecco’s Modified Eagle’s Medium (DMEM; Gibco, USA) supplemented with 10% fetal bovine serum (FBS; Gibco, USA), 1% penicillin-streptomycin (Thermo Fisher Scientific), 1% L-glutamine (Thermo Fisher Scientific), and 1% non-essential amino acids (Thermo Fisher Scientific) at 37°C with 5% CO₂. FreeStyle CHO-S suspension cells (Invitrogen, USA) were cultured in EX-CELL 325 PF CHO Serum-Free Medium (Sigma-Aldrich) supplemented with 1% L-glutamine and 1% penicillin-streptomycin. Cells were maintained in nonpyrogenic, vented polycarbonate Erlenmeyer flasks (Thermo Fisher Scientific) at 37°C with 5% CO₂ and constant shaking at 130 rpm in an incubator shaker. All SARS-CoV-2 viruses were isolated from nasopharyngeal swab samples collected from nucleic acid-positive cases and subsequently propagated and titrated in Vero E6 cells as previously described (36). The viral strains included the following: SARS-CoV-2 ancestral strain (GenBank: MT622319.1), Omicron BA.2 (GenBank: MT627325.1), Omicron BA.5 (GenBank: PQ213095.1), and Omicron BF.7 (GenBank: PQ351184.1).

The SARS-CoV-2 S1 protein, S2 protein, and RBD protein corresponding to the ancestral, Delta, and Omicron variants were obtained from Sino Biological (Beijing, China).

### Spike protein expression

The mammalian cell codon-optimized ectodomain of ancestral SARS-CoV-2 spike trimer cDNA was synthesized by Generay Biotech Co., Ltd (Shanghai, China), in which a trimeric foldon from T4 phage fibritin (Tfd) was fused to the C-terminus of SARS-CoV-2 spike ectodomain (residues 1–1,211; GenBank: NC_045512.2), with the furin cleavage site RRAR replaced by GSAS and two proline mutations (K986P/V987P) introduced to stabilize the prefusion conformation, along with a C-terminal polyhistidine tag for purification (1). This cDNA was cloned into a modified pCI-GS CHO expression vector and electroporated into CHO cells, followed by selection of stable spike trimer-expressing clones using glutamine-deficient medium containing 25 µM methionine sulfoximine (Sigma). A selected clone was gradually scaled up to 200 mL culture volume, after which supernatants were harvested and purified sequentially through ultrafiltration, nickel affinity chromatography, and desalting. The purified recombinant protein (designated S-2P) was quantified by BCA assay (Thermo Fisher Scientific), with purity verified by Coomassie blue staining and S1-domain-specific western blotting under both denaturing and non-denaturing conditions.

### Assay of S-2P binding to human ACE2

The binding of S-2P to human ACE2 was assessed by enzyme-linked immunosorbent assay (ELISA). Briefly, S-2P protein (2 µg/mL) was coated onto high-binding 96-well plates (Thermo Fisher Scientific) and incubated overnight at 4°C. After washing with PBST (PBS containing 1% Tween-20), plates were blocked with blocking buffer (PBST containing 1% BSA) for 2 hours at room temperature. Following another PBST wash, serially diluted human ACE2 protein (mFc Tag; Sino Biological, Beijing, China) was added to the plates and incubated for 2 hours at room temperature. After washing, HRP-conjugated goat anti-mouse IgG1 cross-adsorbed secondary antibody (Invitrogen; 1:1,000 dilution) was added and incubated for 2 hours at room temperature. Following final PBST washes, the reaction was developed using 1-Step Slow TMB solution (Thermo Fisher Scientific) and stopped with ELISA Stop Solution (Solarbio, China). Absorbance was measured at 450 nm.

### Animals and ethics statement

C57BL/6 mice were obtained from Shanghai Jihui Laboratory Animal Care Co., Ltd. (Shanghai, China), and CAG-hACE2 transgenic mice on the C57BL/6 background were acquired from Shanghai Model Organisms Center, Inc. (Shanghai, China). Unless otherwise specified, the experimental mice were 6–8 weeks of age. For the aged group, 18-month-old mice were used, as the standard laboratory mouse has an average lifespan of about 2 years, with 18–24 months generally considered old age (37). All animals were housed in individually ventilated cages under specific pathogen-free conditions. All animal experiments were reviewed and approved by the Institutional Committee on Ethics of Medicine of Navy Medical University and conducted in accordance with China’s Regulations for the Administration of Affairs Concerning Experimental Animals.

### Mouse immunization

The recombinant S-2P protein was formulated with alum (Croda, Denmark), Montanide ISA 720 VG, Montanide ISA 51 VG, or Sepivac SWE (all from Seppic, France), designated as S-2P:Al, S-2P:ISA720, S-2P:ISA51, and S-2P:SWE, respectively. Each mouse was intramuscularly injected with 100 µL of vaccine formulation containing 10 µg of S-2P protein, with adjuvant volume ratios of 70%, 50%, and 50% for S-2P:ISA720, S-2P:ISA51, and S-2P:SWE, respectively, while S-2P:Al contained 100 µg of aluminum hydroxide. Unless otherwise specified, C57BL/6J or hACE2 mice were immunized twice at 3 week intervals with S-2P:Al, S-2P:ISA720, S-2P:SWE, or S-2P:ISA51, while control animals received PBS alone.

### Serum antibody assay

Mouse serum antibodies against SARS-CoV-2 were evaluated by ELISA as described (38). Briefly, recombinant SARS-CoV-2 S1, S2, or RBD protein (1 µg/mL) was coated onto high-binding 96-well plates overnight at 4°C, followed by blocking with blocking buffer. Serially diluted serum samples were then added to the plates and incubated at room temperature for 2 hours. After extensive washing, HRP-conjugated secondary antibodies (goat anti-mouse IgG, goat anti-mouse IgG1, or goat anti-mouse IgG2b; Thermo Fisher Scientific) were added. Endpoint titers were determined as the reciprocal of the highest dilution showing an absorbance at 450 nm ≥2.1fold higher than control mouse sera.

For the blocking ELISA assessing antibody inhibition of RBD-hACE2 binding, RBD-coated (1 µg/mL) 96-well plates were first incubated with serially diluted serum samples for 2 hours at room temperature before adding hACE2 protein (0.25 µg/mL). After washing, rabbit anti-hACE2 antibody (Sino Biological) was added for 2 hours at room temperature, followed by HRP-conjugated goat anti-rabbit IgG (Thermo Fisher Scientific). The remaining steps were performed as described above.

### Serum neutralization of authentic SARS-CoV-2

Vero E6 cells were seeded in 96-well plates (1 × 10⁴ Vero E6 cells per well) and cultured overnight. Serially diluted serum samples were mixed with either 0.1 MOI (multiplicity of infection) of SARS-CoV-2 ancestral strain or 0.01 MOI of Omicron BA.2 strain at 37°C for 30 minutes, then the virus-serum mixtures were added to Vero E6 cells in the presence of 2 µg/mL TPCK-treated trypsin. At 24 hours post-infection, cells were fixed with methanol at −20°C for 30 minutes and blocked with 3% BSA. Rabbit polyclonal antibodies against SARS-CoV-2 nucleocapsid protein (NP; Sino Biological, Beijing, China) were added and incubated overnight at 4°C, followed by PBS washing and incubation with Alexa Fluor 488-conjugated goat anti-rabbit IgG (Thermo Fisher Scientific) at room temperature for 1.5 hours. After DAPI staining (Sigma-Aldrich), cells were imaged using a Cytation 5 system (BioTek, USA). Infected cell numbers were quantified using Gen5 3.10 software, with neutralization percentage calculated relative to virus-only controls. The 50% inhibitory concentration (IC₅₀) was determined as the reciprocal serum dilution showing 50% infection inhibition and calculated using GraphPad Prism 8.0 (GraphPad Software, Inc.).

### Serum neutralization of pseudotyped virus

HEK293T cells were co-transfected using Lipofectamine 2000 reagent (Thermo Fisher Scientific) with HIV capsid packaging plasmids, a transfer plasmid harboring the EGFP reporter gene, and a plasmid harboring the spike gene of SARS-CoV-2 or SARS-CoV. In the SARS-CoV-2 spike expression plasmid, the fragment encoding a 19-amino-acid at the C-terminal of spike was truncated. Following 48 hours of incubation, pseudovirus-containing supernatants were harvested, centrifuged at 1,000 × *g* for 10 minutes, and stored at −80°C until use. For neutralization assays, serially diluted samples of the test sera were pre-incubated with pseudoviruses at 37°C with 5% CO₂ for 30 minutes, then the mixtures were added to 293T-ACE2 target cells (seeded at 1 × 10⁴ cells/well). After 4 hours, the medium was replaced with fresh DMEM supplemented with 2% FBS, and EGFP-positive cells were quantified at 48 hours post-infection using a Cytation 5 cell imaging multimode reader (BioTek). Neutralization titers (ID50) were calculated using nonlinear regression analysis in GraphPad Prism 8.0 (GraphPad Software).

### Enzyme-linked immunosorbent spot assay

T-cell-mediated immune responses in mice were evaluated using an interferon gamma (IFN-γ) ELISPOT kit (Dakewe, China) as described (38). Spleen and lung cells were isolated 14 days post-secondary immunization. Briefly, IFN-γ monoclonal antibody-precoated ELISpot plates were blocked with 200 µL/well of serum-free ELISPOT medium (Dakewe, China) for 5–10 minutes at room temperature. Spleen or lung cells (2 × 10⁵/well) were stimulated with ancestral SARS-CoV-2 S1 or S2 protein at 400 µg/mL for 20 hours at 37°C with 5% CO₂. IFN-γ-producing cells were detected using biotinylated detection antibody and streptavidin-HRP, followed by spot visualization with an S6 Ultra ImmunoSpot Reader (Cellular Technology Ltd.) and quantification using ImmunoSpot 5.1.36 software (Cellular Technology Ltd.). Spot-forming cells were enumerated as a measure of antigen-specific T-cell responses.

### Challenge with SARS-CoV-2

Under isoflurane anesthesia, hACE2 transgenic mice were intranasally inoculated with 50 µL of viral suspension containing SARS-CoV-2 ancestral strain (1 × 10² PFU), Omicron BA.5 (1 × 10^4^ PFU), or Omicron BF.7 (1 × 10^4^ PFU) as described (38). Mice were monitored daily for body weight changes and survival rates. At 4 days post-infection (dpi), a subset of mice was humanely euthanized for tissue collection (nasal turbinates, lungs, and brains) to assess viral load and inflammatory factors, while the remaining mice were maintained for longitudinal survival analysis.

### Measurement of viral load and inflammatory cytokines

Viral RNA levels and inflammatory cytokine gene expression were analyzed by quantitative reverse transcription PCR (qRT-PCR). Tissue samples (brain, lung, and nasal turbinates) from mice were homogenized in TRIzol Reagent (Invitrogen) for total RNA extraction according to the manufacturer’s protocol. First-strand cDNA synthesis was performed using PrimeScript RT Master Mix (TaKaRa, Japan), followed by quantitative PCR amplification with TB Green Premix Ex Taq II (TaKaRa, Japan) and the following gene-specific primers (5′→3′): SARS-CoV-2 nucleocapsid (N) gene (forward: AAGGCGTTCCAATTAACACCA, reverse: TGCCGTCTTTGTTAGCACCA); mouse β-actin (forward: GGCTGTATTCCCCTCCATCG, reverse: GCACAGGGTGCTCCTCAG); IL-6 (forward: TCGGAGGCTTAATTACACA, reverse: TCATACAATCAGAATTGCCAT); CCL2 (forward: TCGGAACCAAATGAGATCAGA, reverse: TAGCTTCAGATTTACGGGTCA); and CXCL10 (forward: AATTTAATGAAAGCGTTTAGCC, reverse: ATTAGGACTAGCCATCCAC). All data were normalized to β-actin expression levels.

### Serum adoptive transfer

Passive immunization was performed through adoptive transfer of immune sera to evaluate protective efficacy against SARS-CoV-2 challenge in recipient hACE2-transgenic mice, as previously described (39). Briefly, hACE2 transgenic mice were intravenously administered 0.1 mL of pooled sera collected 14 days post-booster immunization (from vaccinated mice) or PBS-treated control mice. Twenty-four hours post-serum transfer, mice were intranasally challenged with live SARS-CoV-2 ancestral strain. Body weights were monitored daily until day 4 post-infection, when mice were humanely euthanized for virological and immunological analyses. Viral RNA loads and inflammatory factor expression profiles in brain tissues were quantified by qRT-PCR.

### Statistical analysis

Statistical analyses were conducted using GraphPad Prism 8.0 (GraphPad Software). Between-group comparisons were performed using either unpaired or paired two-tailed Student’s t-tests as appropriate, while multiple group comparisons were analyzed by one-way ANOVA followed by Tukey’s post hoc test. Statistical significance was defined as *P* < 0.05, with the following notation: *P < 0.05, **P < 0.01, and ***P < 0.001; ns (not significant) for *P* ≥ 0.05.

## RESULTS

### Immune responses to ancestral SARS-CoV-2 spike trimers formulated with different human-compatible adjuvants in C57BL/6 mice

The prefusion-stabilized spike trimer of ancestral SARS-CoV-2 was expressed in CHO cells and purified via affinity chromatography. The purified protein, designated S-2P, was confirmed by native and denaturing SDS-PAGE and western blot using a monoclonal antibody (mAb) targeting the S1 subunit (Fig. S1A). ELISA analysis demonstrated dose-dependent binding of soluble human ACE2 to immobilized S-2P (Fig. S1B). Furthermore, S-2P effectively inhibited SARS-CoV-2 infection in Vero-E6 cells (Fig. S1C).

**Fig 1:**
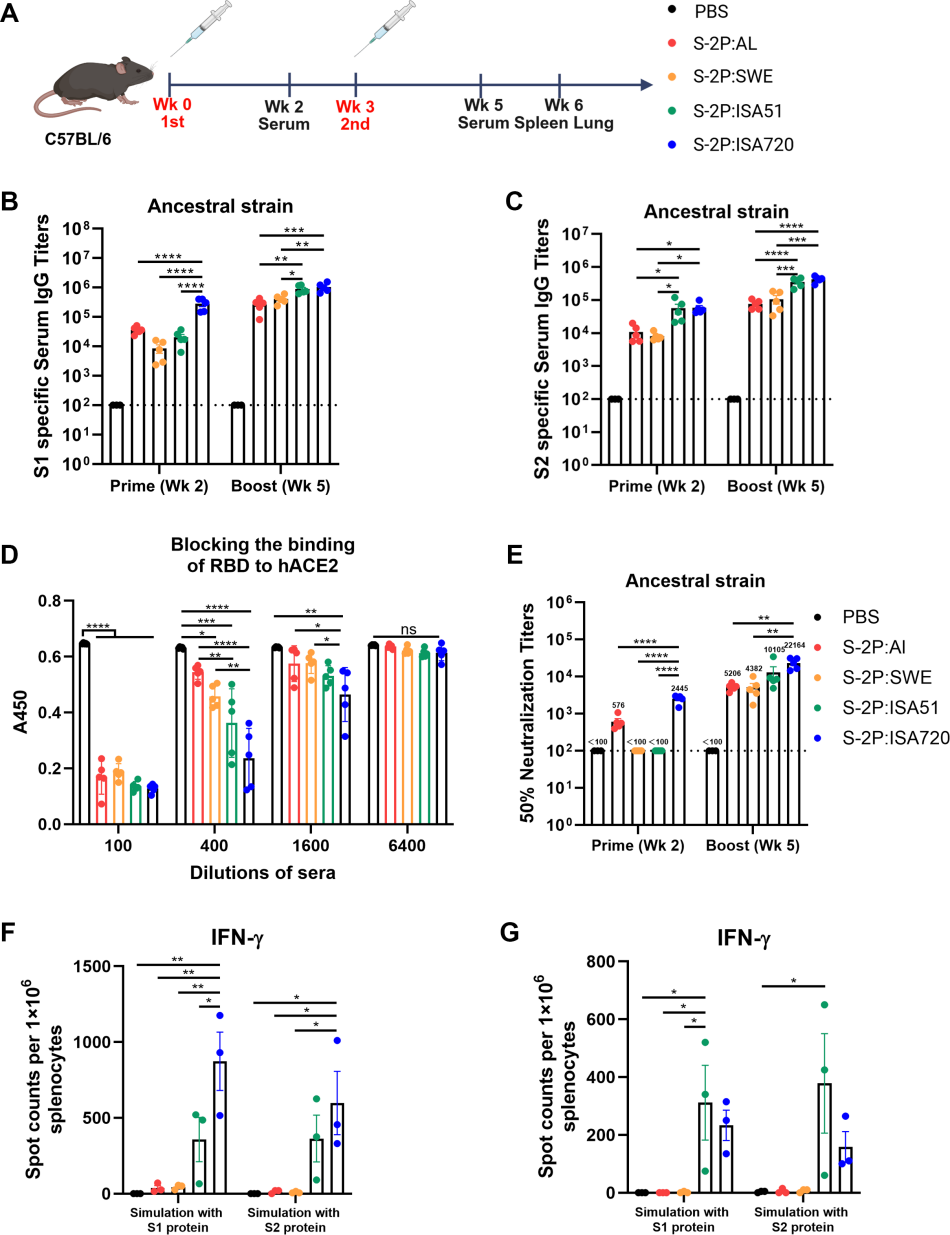
Immunogenicity evaluation of adjuvanted ancestral SARS-CoV-2 S-2P vaccines in C57BL/6 mice. (**A**) Scheme of immunization and sampling of C57BL/6 mice (*n* = 5). The mice were intramuscularly administered with 10 µg S-2P protein formulated with different adjuvants per dose following a prime-boost regimen with a 3-week interval. (**B and C**) Serum samples collected at weeks 2 and 5 post-prime immunization were analyzed by ELISA for anti-S1 IgG (**B**) and anti-S2 IgG titers (**C**) (*n* = 5 mice/group). Titers are expressed as the reciprocal of the endpoint serum dilution (log10 scale). The dashed line indicates the lower limit of detection (100-fold dilution); samples below detection were assigned a titer of 100. (**D**) The serum collected at week 5 was assessed for the ability to block hACE2 binding to SARS-CoV-2 RBD by competitive ELISA (*n* = 5). (**E**) The serum collected at week 5 post-prime immunization was evaluated for neutralizing antibody titers (NT50) against authentic SARS-CoV-2 (*n* = 5). The dashed line indicates the lowest detection limit (NT50 = 100). (**F and G**) Splenocytes and pneumonocytes were isolated at week 6 post-prime immunization and stimulated with S1 or S2 protein; the IFN-γ-producing cells were quantified by ELISpot (*n* = 3). Statistical analysis: Data represent mean ± SEM. Significance was determined by one-way ANOVA with multiple comparisons test.

Aluminum hydroxide (Croda, Denmark) and Seppic’s Montanide ISA 720, ISA 51, and Sepivac SWE (Seppic, France) were evaluated for their ability to enhance immune responses to recombinant S-2P protein in C57BL/6 mice. Mice were immunized intramuscularly twice at a 3-week interval with 10 µg S-2P protein formulated with either aluminum hydroxide or Seppic’s adjuvants (denoted as S-2P:Al, S-2P:ISA720, S-2P:ISA51, and S-2P:SWE, respectively). Serum samples were collected 2 weeks after each immunization and analyzed for antigen-specific IgG and neutralizing antibodies (Fig. 1A). Comparative analysis showed significant differences in immunogenicity depending on the adjuvant used. Both S-2P:ISA720 and S-2P:ISA51 induced stronger antibody responses compared to S-2P:Al or S-2P:SWE. Sera from S-2P:ISA720-immunized mice showed the highest levels of S1- and S2-specific IgG (Fig. 1B and C), the strongest inhibition of RBD binding to hACE2 (Fig. 1D) and the most potent neutralizing activity against the ancestral SARS-CoV-2 (4.3-, 5.1- and 2.2-fold higher than S-2P:Al, S-2P:SWE and S-2P:ISA51 groups at week 5, respectively) (Fig. 1E).

To assess antigen-specific T-cell responses, immunized mice were sacrificed 3 weeks after the booster dose. Single-cell suspensions were prepared from both spleens and lungs, then stimulated *ex vivo* with ancestral S1 or S2 proteins. IFN-γ-producing cells were quantified using enzyme-linked immunospot (ELISpot) assay. The results showed that mice immunized with S-2P:ISA720 or S-2P:ISA51 exhibited significantly stronger antigen-specific T-cell responses in both splenic and pulmonary compartments, whereas S-2P:Al and S-2P:SWE failed to induce detectable antigen-specific IFN-γ-producing T cells (Fig. 1E and F; Fig. S1D and E).

### Adjuvant-dependent protection of S-2P vaccines against lethal SARS-CoV-2 challenge in humanized ACE2 mouse model

**Fig 2:**
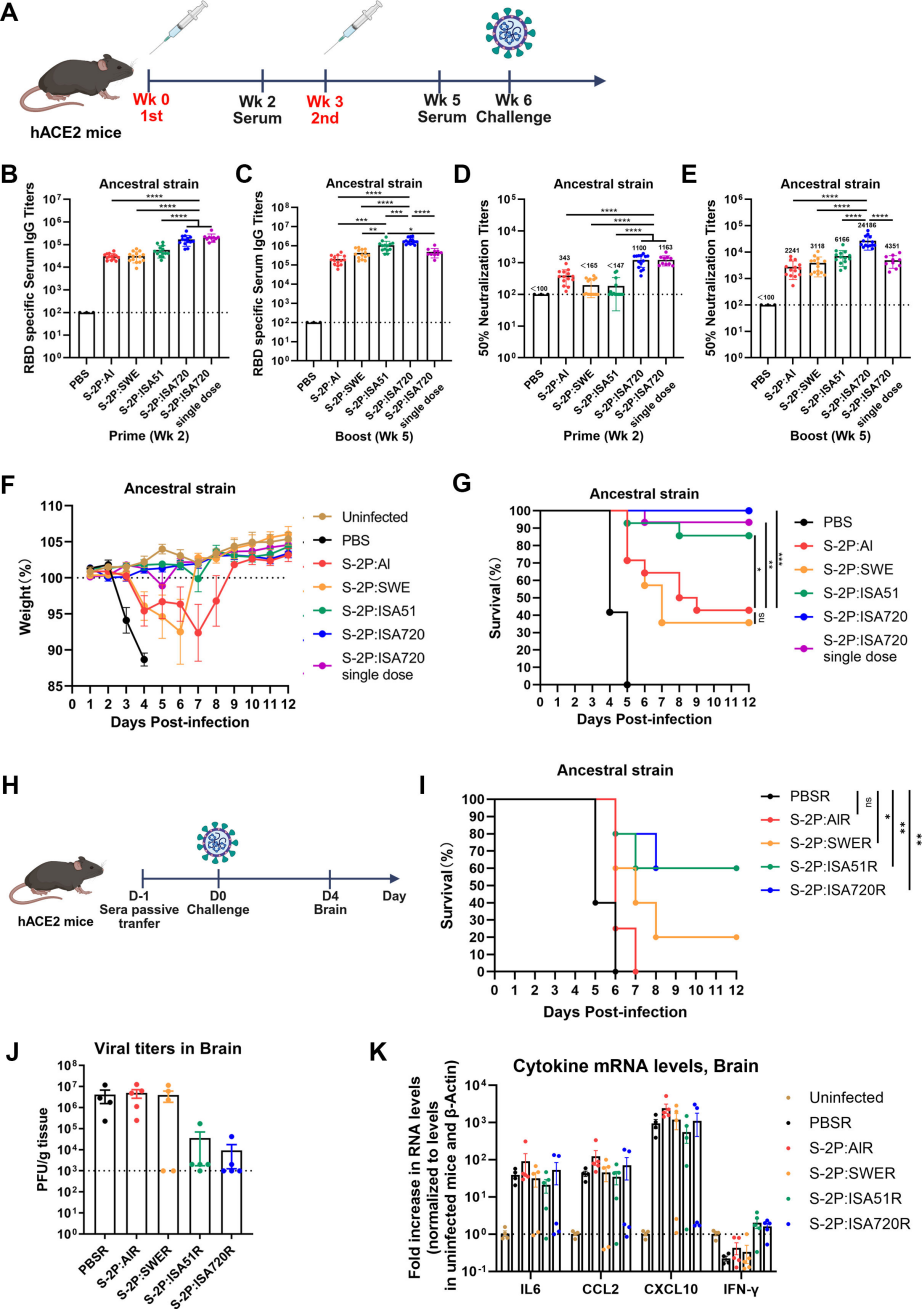
Protective efficacy of vaccine candidates against lethal SARS-CoV-2 challenge in hACE2 mice. (**A**) Experimental schedule. The transgenic hACE2-C57BL/6 mice (*n* = 14/group) were intramuscularly administered with different adjuvanted S-2P formulations following a prime-boost regimen with a 3-week interval plus an additional single-dose S-2P:ISA720 group (*n* = 10). At week 6 post-prime, mice were challenged intranasally with ancestral SARS-CoV-2. (**B and C**) Anti-RBD IgG endpoint titers at weeks 2 and 5 by ELISA (*n* = 10-14). (**D and E**) Neutralizing antibody titers (NT50) against authentic virus (*n* = 10–14). (**F and G**) Daily body weight changes (%) and survival curves post-challenge. (**H**) Experimental schedule. Naïve hACE2 mice received 100 µL pooled sera from immunized or PBS-control mice (*n* = 10/group) one day before SARS-CoV-2 challenge. Five mice per group were sacrificed at 4 days post-infection for analysis of brain viral loads and cytokine profiles, while the remaining five mice were monitored for clinical outcomes. (**I**) Survival curves of recipient mice following SARS-CoV-2 challenge. (**J**) Brain viral loads by plaque assay. (**K**) Brain inflammatory cytokine mRNA levels by RT-qPCR assay. Data shown as mean ± SEM. Statistics: one-way ANOVA with Tukey’s multiple comparisons test.

The CAG-hACE2 transgenic mouse represents a valuable small animal model for evaluating candidate vaccine efficacy. Although SARS-CoV-2 infection affects both upper and lower respiratory tracts and induces tissue damage, viral invasion into the central nervous system emerges as the primary cause of mortality in this model (40). We immunized CAG-hACE2 transgenic mice (C57BL/6 background) twice at 3-week intervals with 10 µg S-2P protein formulated with different adjuvants (14 mice/group), along with an additional S-2P:ISA720 single-dose group (10 mice) (Fig. 2A). Consistent with observations in wild-type C57BL/6 mice, all formulations efficiently induced anti-RBD IgG (Fig. 2B and C) and virus-neutralizing antibodies in hACE2 mice (Fig. 2D and E), with S-2P:ISA720 showing superior immunogenicity, exhibiting 10.8-, 7.8-, and 3.9-fold higher neutralizing titers versus S-2P:Al, S-2P:SWE, and S-2P:ISA51, respectively at week 5. Remarkably, single-dose S-2P:ISA720 outperformed two-dose S-2P:Al and S-2P:SWE in neutralizing antibody induction, while S-2P:Al showed consistently weaker responses than ISA51- or ISA720-formulated vaccines (Fig. 2C and E). Moreover, S-2P:ISA51 and S-2P:ISA720 induced Th1-skewed immunity (evidenced by elevated IgG2b:IgG1 ratios; Fig. S2A and B) that correlated with a robust IFN-γ-producing T-cell response observed in wild-type C57BL/6 mice.

Three weeks after booster immunization, mice were intranasally challenged with 100 PFU of ancestral SARS-CoV-2. Unvaccinated control mice exhibited rapid disease progression, with severe weight loss and 100% mortality within 5 days post-challenge (Fig. 2F and G). All vaccinated groups showed varying degrees of protection: the S-2P:ISA720 group demonstrated complete protection (100% survival, 14/14), while S-2P:ISA51 showed 85.7% survival (12/14) (Fig. 2F and G). Comparatively, S-2P:SWE and S-2P:Al groups exhibited relatively lower protection rates of 42.9% (6/14) and 35.7% (5/14), respectively. Notably, even a single dose of S-2P:ISA720 conferred 90% protection (9/10 survivors), highlighting its superior efficacy.

To elucidate the protective role of vaccine-induced neutralizing antibodies, we analyzed the correlation between humoral immune responses and survival rates in vaccinated mice. The S-2P:Al and S-2P:ISA720 groups demonstrated strong correlations between anti-RBD IgG and neutralizing antibody titers, while the S-2P:SWE and S-2P:ISA51 group showed a relatively weaker correlation (Fig. S2C through G), suggesting adjuvants critically influence both the magnitude and functional quality of antibody responses. Protection analysis revealed that neutralizing antibody titers ≧3 × 10^3^ conferred complete protection (5/5) in S-2P:Al-immunized mice and 71% protection (5/7) in S-2P:SWE-immunized mice (Fig. S2C through G). Since neither S-2P:Al nor S-2P:SWE immunization induced detectable T-cell response, the observed protection can be primarily attributed to neutralizing antibodies.

We further confirmed the protective efficacy of vaccine-induced antibodies through passive immunization in naïve hACE2 mice. The mice (*n* = 10 per group) were administered 100 µL of pooled sera from immunized or control mice 1 day prior to SARS-CoV-2 challenge. Five mice per group were sacrificed at 4 days post-infection for analysis of brain viral loads and cytokine profiles, while the remaining five mice were monitored for clinical outcomes. All mice that received control sera or sera from S-2P:Al-immunized mice exhibited significant weight loss and mortality. In contrast, Seppic-adjuvanted vaccine immune sera conferred partial protection, with 1, 3, and 3 mice receiving sera from mice immunized with SWE-, ISA51-, and ISA720-adjuvanted vaccine surviving, respectively (Fig. 2H). The observed reduction in viral titers and pro-inflammatory cytokine levels in murine brain tissue demonstrates that vaccine-induced antibodies effectively inhibit SARS-CoV-2 neuroinvasion and mitigate associated neuropathological damage (Fig. 2I and J).

### ISA720-adjuvanted ancestral S-2P conferred complete protection against challenge with Omicron variants

Pseudovirus-based neutralization assays were employed to quantify the cross-neutralizing antibody induced by various adjuvanted S-2P vaccines against SARS-CoV-2 variants of concern (VOCs) in murine models. While Delta variant neutralization showed modest (0.8- to 1.3-fold) titer reduction versus ancestral strain, Omicron BA.1 neutralization was substantially impaired across sera of all groups (Fig. S3A). The majority of sera from S-2P:Al- and S-2P:SWE-immunized mice showed no detectable neutralization against BA.1 at 100-fold dilution, while 84.6% (11/13) of S-2P:ISA51 and 100% (11/11) of S-2P:ISA720 immune sera maintained neutralizing activity, despite a 17-fold decrease in geometric mean neutralization titers compared to neutralization against ancestral strain for the S-2P:ISA720 group. Importantly, both ISA-adjuvanted vaccine groups demonstrated cross-neutralization capacity against SARS-CoV-1 pseudovirus at low serum dilutions (Fig. S3B).

**Fig 3:**
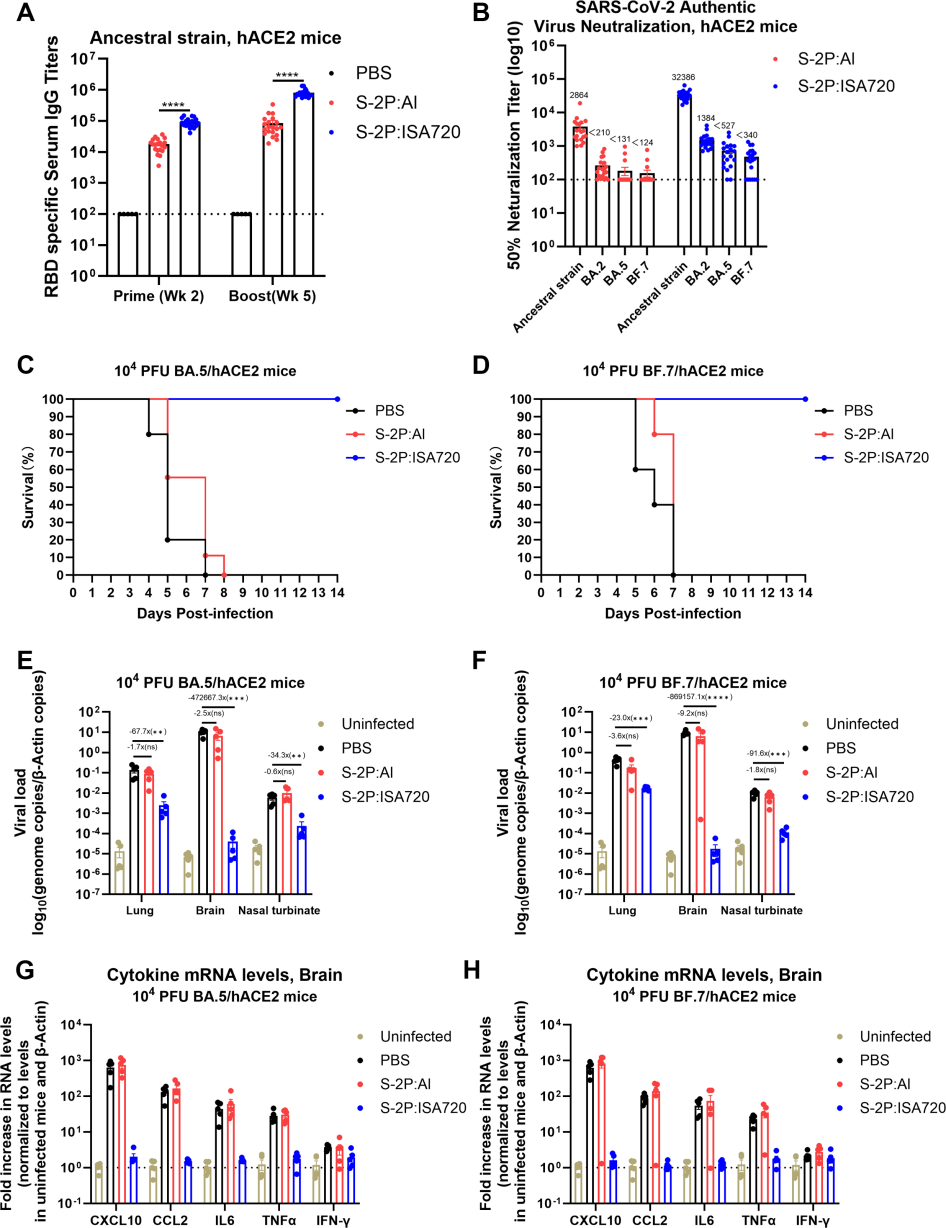
Protective efficacy of S-2P:Al versus S-2P:ISA720 against Omicron BA.5/BF.7 variants. The hACE2 mice (*n* = 10 per group) received prime and boost immunization with S-2P:Al or S-2P:ISA720 at weeks 0 and 3. Sera were collected at weeks 2 and 5. Three weeks post-boost immunization, mice were challenged with either Omicron BA.5 or BF.7 variants. Five mice per group were monitored for clinical outcomes, and the remaining five mice were sacrificed at 4 days post-infection for analysis of viral nucleic acid loads and mRNA levels of inflammatory factors. Serum was collected at weeks 2 and 5 post-prime. The mice were challenged with either Omicron BA.5 or BF.7 variants at week 3 post-boost. Five mice per group were monitored for clinical outcomes, and the remaining five mice were sacrificed at 4 days post-infection for analysis of viral nucleic acid loads and inflammatory factor mRNAs. (**A**) Anti-ancestral RBD IgG endpoint titers at weeks 2 and 5 by ELISA. (**B**) Serum neutralization (IC50) against authentic viruses of ancestral strain and Omicron VOCs (BA.2, BA.5, and BF.7) at week 5. (**C and D**) Body weight changes (%) and survival kinetics. (**E and F**) Viral RNA loads in lung, brain, and nasal turbinate assessed by RT-qPCR assay. (**G and H**) Brain inflammatory cytokines mRNA levels assessed by RT-qPCR assay. Data are represented as the mean ± SEM. Groups were compared using one-way ANOVA with Tukey’s multiple comparisons test.

Next, hACE2-transgenic mice were immunized with either S-2P:Al or S-2P:ISA720 to evaluate the *in vivo* protective efficacy against Omicron variants BA.5 and BF.7. Consistent with the findings in Results 1 and 2, immunization with S-2P:ISA720 elicited significantly higher titers of anti-RBD antibody and neutralizing antibody against the ancestral strain compared to S-2P:Al (Fig. 3A and B). Notably, in authentic virus neutralization assays, sera from the majority of S-2P:ISA720-immunized mice potently neutralized Omicron subvariants BA.2, BA.5, and BF.7. In contrast, only minimal neutralizing activity was detected in sera from S-2P:Al-immunized mice, with significantly lower titers (Fig. 3B). Following viral challenge, all of the S-2P:Al-immunized mice exhibited progressive weight loss and succumbed (Fig. 3C and D; Fig. S3C and D). Conversely, all of the S-2P:ISA720-immunized mice maintained normal body weight and survived (Fig. 3C and D; Fig. S3C and D). Quantitative analysis demonstrated that S-2P:ISA720 vaccination significantly reduced viral loads in lungs, brains, and nasal turbinates (Fig. 3E and F). Correspondingly, cytokine mRNA profiling in brain tissues revealed marked inflammatory responses in PBS-control and S-2P:Al groups, but minimal response in S-2P:ISA720-immunized mice (Fig. 3G and H).

In summary, ISA720-adjuvanted S-2P not only broadened the neutralization capacity against diverse SARS-CoV-2 variants but, more significantly, conferred robust cross-protection against antigenically distinct VOCs.

### Sustained ceiling-level antibody responses and durable protection following ISA720-adjuvanted S-2P prime-boost vaccination

**Fig 4:**
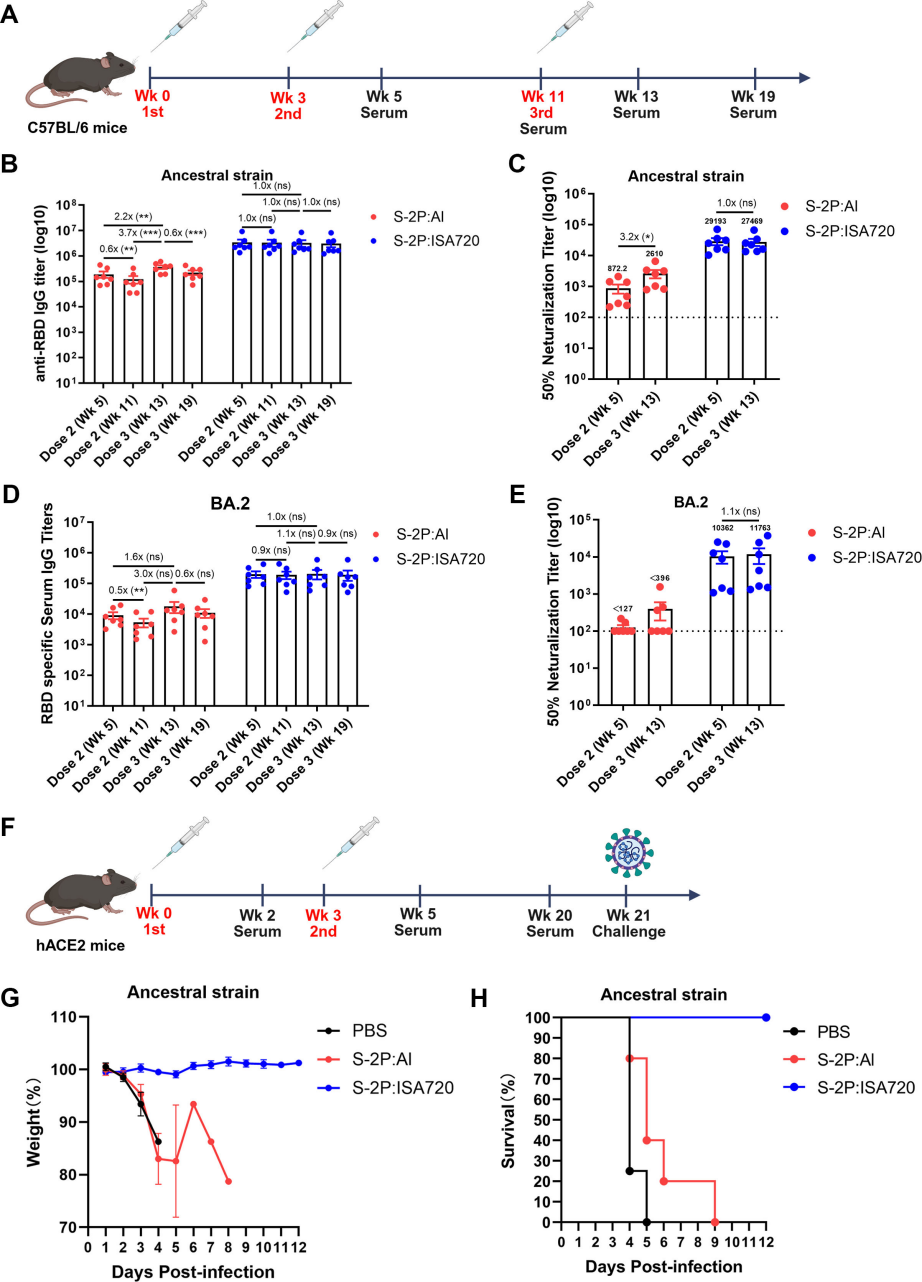
Adjuvant effects on antibody response to booster dose and long-lasting protection. (**A**) The C57BL/6 mice were immunized with S-2P:Al or S-2P:ISA720 three times at weeks 0, 3, and 11. The serum samples were collected at 2 and 8 weeks after the second and third immunizations (weeks 5, 11, 13, and 19), the IgG and neutralizing antibody against ancestral and Omicron BA.2 strains were assayed. (**B and C**) Anti-RBD IgG and neutralizing antibody titers against ancestral SARS-CoV-2. (**D and E**) Anti-RBD IgG and neutralizing antibody titers against Omicron BA.2. (**F**) The hACE2 mice were immunized with S-2P:Al or S-2P:ISA720 following a prime-boost regime at weeks 0 and 3, serum samples were collected at weeks 2, 5, and 20 for antibody assay. The mice were challenged with ancestral SARS-CoV-2 at week 21 and monitored for weight change and survival. (**G and H**) Body weight changes (**G**) and survival curves (**H**) post-challenge. Data shown as mean ± SEM. Groups were compared using one-way ANOVA with Tukey’s multiple comparisons test or unpaired two-sided Student’s t-test.

Booster immunization, whether homologous or heterologous, is a widely adopted strategy to enhance vaccine efficacy against rapidly evolving SARS-CoV-2 variants (20). To assess the impact of booster doses on antibody responses elicited by differently adjuvanted spike proteins, we immunized hACE2 mice three times (weeks 0, 3, and 11) with either S-2P:Al or S-2P:ISA720. Serum samples were collected at 2 and 8 weeks after the second and third immunizations (weeks 5, 11, 13, and 19), enabling a longitudinal assessment of antibody response kinetics (Fig. 4A).

Consistent with Results 1 and 2, S-2P:ISA720 induced significantly higher RBD-specific IgG and neutralizing antibody titers against the ancestral strain than S-2P:Al at all indicated timepoints (Fig. 4B). After the second and third immunizations, anti-RBD IgG antibody titers in S-2P:Al-immunized mice showed faster decay kinetics compared to the S-2P:ISA720 group, indicating superior durability of the S-2P:ISA720-induced humoral response. Notably, while the S-2P:Al group showed a significant increase in IgG titers after the third dose, the S-2P:ISA720 group reached peak IgG levels after two doses, with no further increase after the third dose (Fig. 4B). Similarly, the third dose of S-2P:Al markedly enhanced neutralizing antibody titers against the ancestral strain, whereas the third dose S-2P:ISA720 showed no significant improvement in serum neutralization post-booster (Fig. 4C). These results suggest that antibody responses to S-2P:ISA720 vaccination reached a ceiling effect after prime-boost immunization. Moreover, the S-2P:Al group showed a modest (1.6-fold) increase in IgG antibodies against the Omicron BA.2 RBD after the third dose, while S-2P:ISA720 failed to enhance RBD-specific IgG titers post-booster (Fig. 4D). Neutralization of Omicron BA.2 improved slightly with the third S-2P:Al dose, with the number of sera exhibiting neutralizing titers > 100 increasing from 2 to 3. In contrast, sera from S-2P:ISA720-immunized mice displayed stronger neutralization against Omicron BA.2 after the second dose, but the third dose provided no additional benefit (Fig. 4E).

To evaluate long-term immunity, we compared antibody persistence and protective efficacy between the S-2P:Al and S-2P:ISA720 in hACE2 mice immunized at weeks 0 and 3. The S-2P:Al group exhibited a 6.3-fold decline in anti-RBD IgG titers between weeks 5 and 20, compared to only a 2.4-fold decline in the S-2P:ISA720 group (Fig. S4A). Even worse, the S-2P:Al group showed a more than 7.6-fold decline in neutralizing titers, whereas the S-2P:ISA720 group exhibited only a 2.7-fold decline (Fig. S4B). Following the challenge at week 21 with ancestral SARS-CoV-2, all S-2P:Al-immunized mice ultimately succumbed (albeit with delayed mortality versus PBS controls), whereas the S-2P:ISA720 group achieved complete protection (Fig. 4G and H).

### ISA720-adjuvanted S-2P provided superior protective efficacy compared to Alum-adjuvanted S-2P in aged mice

**Fig 5:**
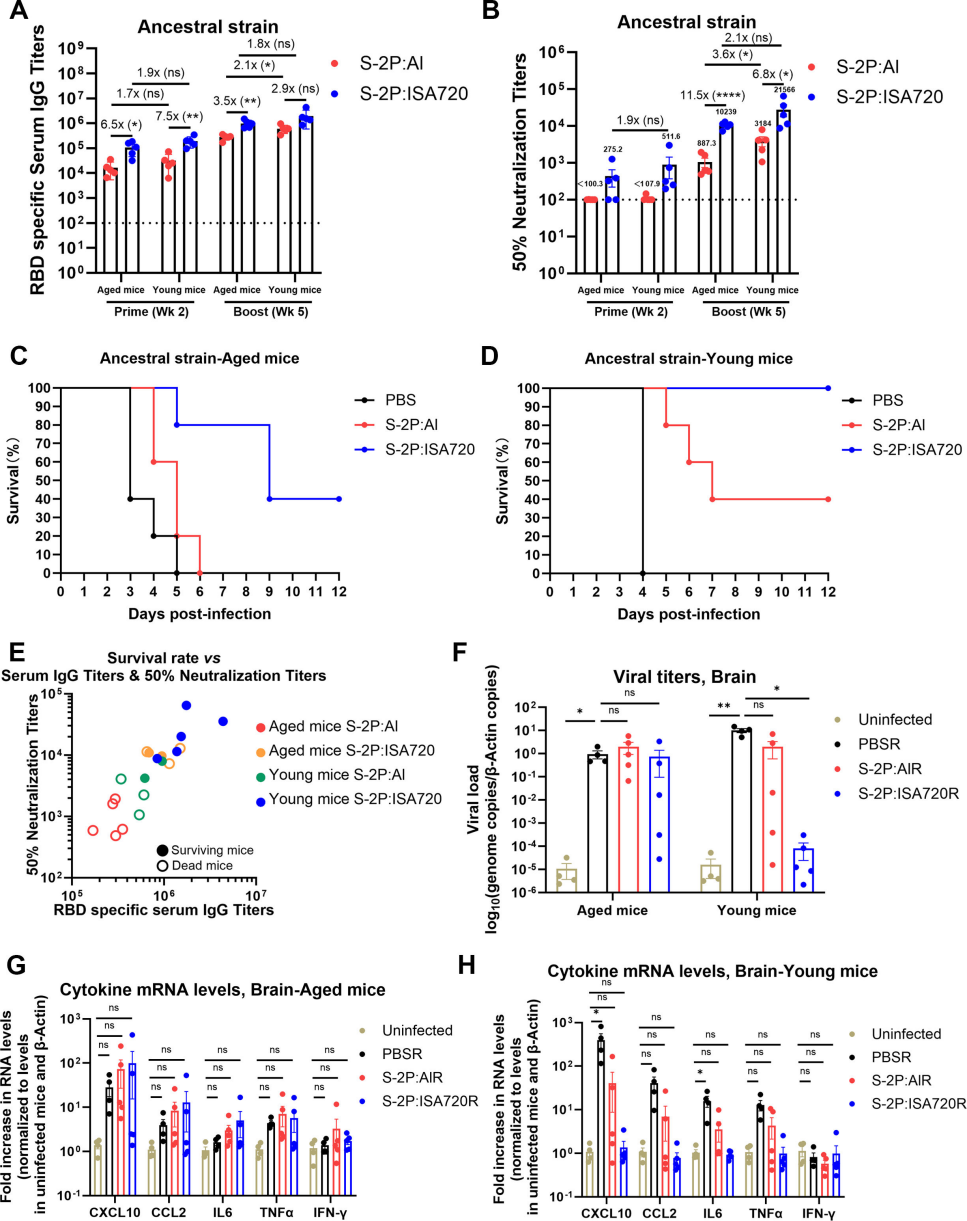
Comparison of vaccine protection between aged and young mice. The hACE2 mice aged 3 and 18 months (*n* = 5/group) received prime-boost immunization with either S-2P:Al or S-2P:ISA720 at a 3-week interval. Serum was collected at weeks 2 and 5, followed by SARS-CoV-2 challenge 3 weeks post-boost. (**A**) Anti-RBD IgG endpoint titers at weeks 2 and 5 by ELISA (*n* = 5). Dashed line indicates 100-fold dilution limit (titer = 100 for undetectable samples). (**B**) Serum neutralization (IC50) against authentic SARS-CoV-2 (*n* = 5) at week 5. Dashed line marks detection threshold (IC50 = 100). (**C and D**) Survival kinetics post-challenge of aged (**C**) and young mice (**D**). (**E**) Mouse serum anti-RBD antibody titers and corresponding neutralizing antibody titers. (**F-H**) Naïve 3- and 18-month-old hACE2 mice received 100 µL pooled sera from immunized or PBS-control mice (*n* = 5/group) one day before SARS-CoV-2 challenge. Mice were euthanized at 4 days post-infection (dpi) for quantitative analysis of viral loads and inflammatory cytokine levels in the brain. (**F**) Brain viral RNA levels assessed by RT-qPCR assay. (**G and H**) Brain inflammatory factors mRNA expression of aged (**G**) and young mice (**H**) assessed by RT-qPCR assay. Data are represented as the mean ± SEM. Groups were compared using one-way ANOVA with Tukey’s multiple comparisons test, except for G and H. The statistical analysis of the data presented in G and H was performed using the Kruskal-Wallis test. Two groups were compared using Student’s t-test, paired or unpaired as indicated.

The elderly population exhibits heightened susceptibility to severe COVID-19 outcomes (17, 18). To evaluate vaccine protection in aging populations, we compared immune responses in young (3-month-old) and aged (18-month-old) hACE2 mice immunized with either S-2P:Al or S-2P:ISA720. Serum antibody responses were measured at 2 weeks after prime and booster vaccination. Following prime immunization, aged mice exhibited significantly lower anti-RBD IgG levels compared to young adults (1.7-fold lower for S-2P:Al and 1.9-fold lower for S-2P:ISA720). This age-dependent reduction persisted after booster immunization, with aged mice showing 2.1-fold (S-2P:Al) and 1.8-fold (S-2P:ISA720) lower antibody levels than their younger counterparts (Fig. 5A). Neutralizing antibody levels after the booster immunization exhibited a similar pattern, being 3.6-fold lower in aged S-2P:Al recipients and 2.1-fold lower in S-2P:ISA720 recipients compared to young adults (Fig. 5B). Consistent with observations in young mice, S-2P:ISA720 similarly elicited a Th1-skewed immune response in aged animals, as demonstrated by significantly elevated IgG2b:IgG1 ratios (Fig. 5A and B). Following SARS-CoV-2 challenge, control and S-2P:Al-immunized aged mice showed rapid weight loss and 100% mortality. In contrast, aged mice immunized with S-2P:ISA720 demonstrated prolonged survival and a 40% survival rate (Fig. 5C and D). These findings demonstrate that while both vaccines displayed substantially reduced efficacy in aged mice, S-2P:ISA720 provided significantly better protection compared with S-2P:Al.

Interestingly, although some aged mice immunized with S-2P:ISA720 developed higher anti-RBD IgG titers than young survivors receiving S-2P:Al immunization, they nevertheless ultimately succumbed to infection (Fig. 5E). To investigate this apparent disconnect between antibody levels and protection in aged animals, we performed passive immunization experiments. Naïve young and aged hACE2 mice received 100 µL of pooled sera from PBS-, S-2P:Al-, or S-2P:ISA720-immunized young donors prior to SARS-CoV-2 challenge. The mice were sacrificed at 4 days post-infection for analysis of brain viral loads and cytokine profiles. Compared to serum from S-2P:Al-vaccinated mice, serum from S-2P:ISA720 vaccinated mice demonstrated superior effect in reducing viral load in both aged and young mouse brains (Fig. 5F). Notably, the antiviral efficacy of serum from either S-2P:Al- or S-2P:ISA720-vaccinated mice was significantly attenuated in aged recipients compared to young recipients, indicating age-dependent impairment of serum-mediated protection. We also observed that young naïve mice exhibited significantly stronger neuroinflammatory responses post-challenge than aged mice, with 14.2-fold higher CXCL10 (*P* = 0.043), 10.3-fold higher CXCL2 (*P* = 0.047), 2.9-fold higher TNF-α (*P* = 0.049), and 9.9-fold elevated IL-6 (*P* = 0.019) levels in brains (Fig. 5G and H). IFN-γ levels showed comparable changes (*P* = 0.126) between age groups. This suggests that the attenuated innate immune response in aged animals may contribute to impaired antibody-mediated protection, ultimately leading to poorer outcomes despite comparable or higher antibody titers.

## DISCUSSION

Recombinant vaccines account for a substantial share of global COVID-19 vaccine supplies. Adjuvants act as indispensable components in mediating effective induction of adaptive immune responses to recombinant vaccines. In this study, we characterized the immune responses induced by recombinant prefusion-stabilized ancestral SARS-CoV-2 spike protein S-2P formulated with aluminum or one of three Seppic emulsion adjuvants (ISA 720, ISA 51, or SWE). Our findings demonstrate that adjuvants critically influence vaccine efficacy across magnitude, breadth, and pattern of immune responses and reveal key insights into the age-dependent decline in vaccine effectiveness as well as the establishment of a sustained vaccination ceiling effect.

Using the ancestral SARS-CoV-2 S-2P protein as antigen, all four adjuvanted vaccines effectively elicited S1-, S2-, and RBD-specific IgG antibodies with neutralizing activity in mice. The S-2P:ISA720 formulation demonstrated superior immunogenicity, closely followed by S-2P:ISA51, with both inducing robust Th1-biased immune responses and strong IFN-γ-producing T-cell responses in both spleen and lung tissues. In contrast, neither S-2P:Al nor S-2P:SWE induced detectable IFN-γ-producing T-cell responses. Two-dose immunization with S-2P:ISA720 conferred complete protection against lethal challenge with homologous SARS-CoV-2, and one dose achieved 90% protection rates, while S-2P:ISA51 also demonstrated excellent protective efficacy. In contrast, alum- and SWE-adjuvanted vaccines exhibited markedly reduced protective effectiveness.

Since alum- and SWE-adjuvanted formulations did not induce detectable T-cell responses, protection was likely mediated primarily by neutralizing antibodies. This is supported by the positive correlation between reduced brain viral loads, decreased brain inflammatory cytokine levels, and higher serum neutralizing antibody titers in individual mice. The protective efficacy of passive transfer of immune sera experiments further underscores the important protective role of vaccine-induced neutralizing antibodies against homologous SARS-CoV-2 infection. It is noteworthy that both SWE- and ISA51-adjuvanted groups exhibited delayed neutralizing antibody activity, potentially due to the lower affinity of antibodies generated during the early immune response. These initial antibodies primarily originate from extrafollicular responses, which typically produce lower-affinity antibodies with diminished neutralizing capacity. Affinity maturation, a process requiring prolonged antigen exposure in germinal center (GC), evolves over time (41, 42). In contrast, the ISA720 adjuvant appears to enhance GC reactions more effectively by activating key cellular components such as follicular helper T (Tfh) cells and follicular dendritic cells (29, 30), thereby facilitating the rapid production of high-affinity antibodies.

VAERD is characterized by pulmonary eosinophil infiltration and enhanced type 2 cytokine responses (28). Recent studies in rodent models have demonstrated VAERD following immunization with SARS-CoV-2 vaccines (28), including inactivated formulations adjuvanted with aluminum hydroxide, which triggered Th2-type inflammatory cytokines and exacerbated respiratory pathology upon heterologous challenge (43). In this study, although key Th2 cytokines such as IL-4 were not quantified, the absence of detectable IFN-γ-producing T-cell responses and a reduced IgG2a/IgG1 ratio in the aluminum adjuvant group collectively suggest a Th2-skewed immune response. By comparing IFN-γ-producing T-cell activity and IgG2a/IgG1 ratios across adjuvant groups, and in light of the essential role of Th1 immunity in vaccine-mediated protection, these findings underscore the translational promise of ISA 720 and ISA 51 adjuvants.

The remarkable ability of SARS-CoV-2 to undergo rapid and continuous evolution has led to recurrent global outbreaks, predominantly driven by Omicron variants (13). Adjuvants are known to enhance the breadth of the immune response to vaccination. For example, AS03 adjuvant induced robust RBD-specific memory B cells with increased cross-neutralization activity (44). In this study, we observed that the magnitude of antibody responses enhanced by various adjuvants correlated with their breadth of neutralization. Sera from hACE2 mice immunized with Alum- or SWE-adjuvanted formulations showed minimal neutralization against the Omicron BA.1 variant, while sera of mice receiving ISA720- or ISA51-adjuvanted formulations demonstrated moderate neutralizing activity. The majority of sera from S-2P:ISA720-immunized mice effectively neutralized multiple Omicron subvariants, whereas only a minority of S-2P:Al immune sera showed detectable activity against these variants. Importantly, vaccination with the ISA720 formulation conferred complete protection against lethal challenge with BA.5 and BF.7 variants, whereas none of the mice receiving the aluminum formulation survived challenge. The significantly reduced viral load and inflammatory cytokine mRNA levels in mouse brains further demonstrated the superior protective efficacy of the ISA720 formulation.

Αlthough S-2P:ISA720 elicited cross-neutralizing antibodies against Omicron variants, their titers were substantially lower than those against the ancestral strain and were also significantly reduced compared to the ancestral-specific antibodies induced by S-2P:Al. Given that both active immunization with S-2P:Al and passive transfer of immune serum conferred partial protection against homologous challenge, the modest cross-neutralizing antibody response induced by S-2P:ISA720 is unlikely to account for the complete protection observed against lethal challenge with Omicron BA.5 and BF.7 variants. By integrating neutralizing antibody titers, T-cell responses, and survival rates after challenge, these results strongly suggest that T-cell immunity serves as the primary mechanism underlying the superior protective efficacy provided by ISA720-based vaccination against Omicron variants. As documented in numerous studies, T-cell epitopes—particularly CD8+ T-cell epitopes—are highly conserved across SARS-CoV-2 variants (16, 39). Moreover, CD8+ T cells have been shown to mediate complete protection independently of neutralizing antibodies (45, 46).

Viral loads were reduced more effectively in the brain than in the nasal turbinates or lungs in S-2P:ISA720 immunized mice upon challenge with Omicron BA.5 and BF.7 variants. This disparity in tissue-specific protection may be attributed to two mechanisms. First, the vaccine does not provide sterilizing immunity but instead limits viral replication. After intranasal challenge, infection is initially established in the respiratory tract, which triggers clonal expansion of memory T cells and a rapid effector response in vaccinated mice. By the time the virus disseminates to the brain, effector T cells have been mobilized and are efficiently recruited to the brain tissue, where they mediate robust antiviral effector functions. Second, strong suppression of viral replication in the respiratory tract of S-2P:ISA720-immunized mice reduces the viral load available to disseminate to the brain.

Consistent with our observation that ISA720 exhibits potent immunostimulatory activity, the AKS-452 vaccine, which comprises an ancestral RBD-human Fc fusion protein adjuvanted with ISA 720, elicited robust neutralizing antibody responses and conferred strong protection in both murine and non-human primate models (47, 48). Furthermore, in a randomized Phase I/II clinical trial, two 45 µg doses administered 28 days apart induced high titers of IgG and broad-spectrum neutralizing antibodies capable of targeting ancestral, Alpha, and Delta variants, alongside sustained IFN-γ-producing T-cell responses that remained detectable through Day 180 (32).

The age-related immunological alterations not only increase susceptibility to severe COVID-19 and mortality, particularly in those with comorbidities, but also impair vaccine responsiveness (49, 50). In this study, we systematically compared the immunogenicity and protective efficacy of Alum- versus ISA720-adjuvanted S-2P vaccines in young adult and aged hACE2 mice. The S-2P:ISA720 formulation elicited significantly higher anti-RBD IgG and neutralizing antibody titers in both age groups relative to S-2P:Al. While both adjuvanted formulations showed age-related reductions in antibody responses, S-2P:Al demonstrated significantly greater decreases in both anti-RBD and neutralizing antibody titers in aged mice compared to S-2P:ISA720. Notably, protection against ancestral SARS-CoV-2 challenge dropped from 40% to 0% in Alum-adjuvanted groups when comparing young versus aged mice, whereas S-2P:ISA720 maintained 40% protection in aged mice. These findings highlight the significant impact of immunosenescence on vaccine efficacy but also pose new challenges for developing vaccination strategies for elderly populations.

Some aged mice received immunization with S-2P:ISA720, despite producing relatively high levels of neutralizing antibodies, still succumbed to SARS-CoV-2 infection, whereas young mice exhibited clear antibody titer-related protection. The experiment of passive transfer of immune sera revealed two additional striking differences between aged and young mice. First, aged mice mounted a substantially attenuated inflammatory cytokine response, characterized by significantly lower production of key chemokines, including CXCL10 and CCL2. During viral infection, CXCL10 and CCL2 often function synergistically to orchestrate immune cell recruitment: CCL2 mediates the initial recruitment of monocytes and macrophages, which, upon activation, produce IFN-γ, which subsequently induces CXCL10 expression. CXCL10 then promotes the recruitment of T cells and NK cells into infected tissues, thereby enhancing the cellular immune response and amplifying antiviral immunity (51). Second, aged mice that received passive antibody transfer also showed significantly reduced protection against viral challenge compared to young recipients. Taken together with the T-cell-mediated protection observed in this study and the well-established role of T cells in vaccine-induced immunity, these findings suggest that the age-related decline in vaccine efficacy is multifactorial in nature. Nonetheless, a substantially impaired innate immune response and the concomitant weakening of cellular immunity likely represent key mechanisms underlying reduced vaccine protection in aged individuals. Additionally, the findings provide new insights into the development of antibody-based immunotherapy strategies against viral infections in the aged population.

Although direct evidence linking neuroinflammation to viral clearance remains limited, these inflammatory mediators are known to enhance antiviral immunity by promoting the recruitment and activation of monocytes and T cells (51). The attenuated neuroinflammatory response observed in aged mice following SARS-CoV-2 infection may therefore compromise viral clearance. Furthermore, accumulating evidence indicates that persistent SARS-CoV-2 components (such as RNA and proteins) in the brain (52), combined with chronic inflammation and immune dysregulation due to sustained SARS-CoV-2 infection (53), can lead to prolonged cytokine production, a mechanism strongly implicated in long COVID. Thus, the diminished neuroinflammatory response in aged mice after viral challenge may contribute to the development of long COVID.

The attenuated inflammatory response observed in aged mice following viral challenge appears to contradict the well-established paradigm of elevated baseline inflammation in elderly populations. By employing young and aged mice as parallel reference groups to analyze post-challenge inflammatory dynamics, our findings reveal distinct age-dependent patterns in inflammatory magnitude, potentially reflecting differential engagement of protective antiviral immunity mechanisms. These results align with recent reports demonstrating similar age-related divergence in inflammatory responses to virus challenge (54, 55).

Booster vaccinations are primarily administered to restore waning antibody levels and broaden immune responses against emerging variants (20). However, repeated immunization may lead to a ceiling effect—a maximum IgG titer, where additional doses fail to elicit significantly higher antibody responses. This immune plateau phenomenon has been well-documented in recipients of frequent influenza vaccines and has similarly been observed in populations receiving multiple SARS-CoV-2 vaccinations (56–58). In this study, two doses of S-2P:ISA720 vaccination induced a ceiling effect, characterized by peak anti-RBD and neutralizing antibody titers against both the homologous strain and heterologous Omicron BA.2 variant that were not enhanced by a third dose. These antibodies sustained at high levels with minimal waning. In contrast, mice immunized with S-2P:Al showed significantly increased antibody titers to ancestral and Omicron BA.2 after the third dose, though the levels remained substantially lower than those achieved with just two doses of S-2P:ISA720. Furthermore, the S-2P:Al group exhibited similar waning kinetics following the third dose as observed after the second dose. The capacity of S-2P:ISA720 to rapidly elicit high antibody titers that plateau satisfies the requirements for durable immune protection. This long-lasting protection was evidenced by 100% survival rates in S-2P:ISA720 immunized mice following viral challenge at 17 weeks post the second dose, while all S-2P:Al-immunized mice succumbed to infection. Our results suggest that adjuvants optimized for both rapid kinetics and broad-spectrum antibody responses could represent a strategic approach for combating continuously evolving SARS-CoV-2.

Collectively, our study demonstrates that optimal adjuvant formulation in recombinant SARS-CoV-2 spike protein vaccine not only significantly enhances the magnitude and breadth of antibody responses but also robustly boosts T-cell immunity. Furthermore, it rapidly induces and sustains peak antibody levels against both the homologous strain and Omicron variants. Notably, aged mice exhibited not only markedly reduced antibody production post-vaccination but also significantly compromised antiviral functionality of the elicited antibodies. These findings provide critical insights for developing next-generation SARS-CoV-2 vaccine strategies tailored for broad and durable protection.

## Data Availability

The data associated with this paper are available upon request to the corresponding author.

## SUPPLEMENTAL MATERIAL

Supplemental figures jvi.01099-25-s0001.docx Figures S1 to S5.

ASM does not own the copyrights to Supplemental Material that may be linked to, or accessed through, an article. The authors have granted ASM a non-exclusive, world-wide license to publish the Supplemental Material files. Please contact the corresponding author directly for reuse.
