# Supplementary material for: Adjuvant-dependent protection of SARS-CoV-2 spike vaccines: comparative immunogenicity of human-applicable formulations
Source: J Virol. 2025 Oct 3;99(10):e01099-25. doi: 10.1128/jvi.01099-25 (PMC12548409; doi:10.1128/jvi.01099-25)
Supplement: Supplemental figures — Figures S1 to S5. [file jvi.01099-25-s0001.docx]

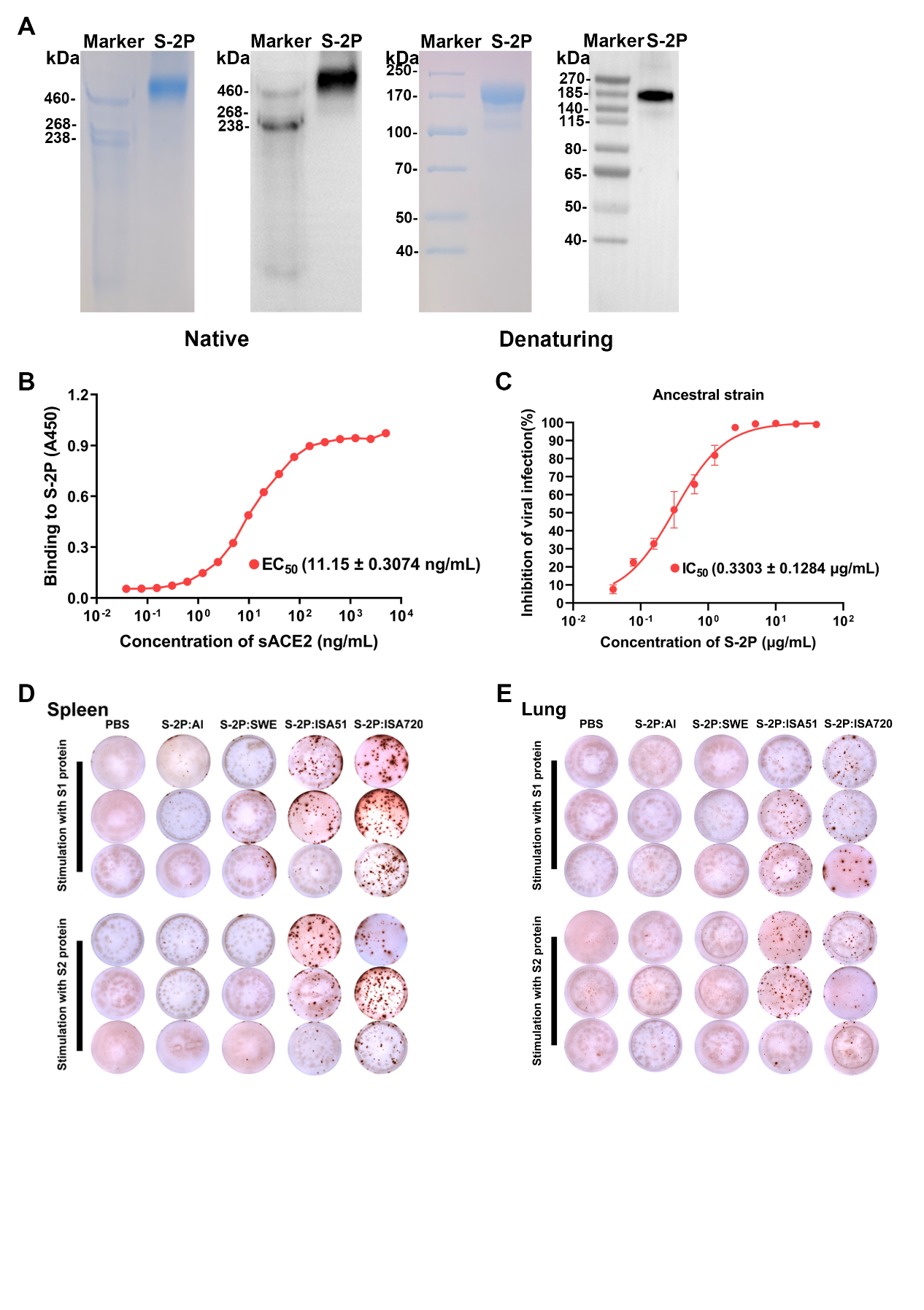


**Fig S1** Biochemical and functional characterization of the prefusion-stabilized ancestral SARS-CoV-2 spike trimer (S-2P) expressed in CHO cells. (A) Purified ancestral SARS-CoV-2 S-2P protein was analyzed by (left) Coomassie-stained SDS-PAGE and (right) western blot under native and denaturing conditions, probed with anti-S1 mAb. (B) Serial dilutions of hACE2 were tested for binding to immobilized S-2P by ELISA. Data are means of triplicate samples from a representative experiment. (C) S-2P dose-dependently inhibited authentic ancestral SARS-CoV-2 infection in Vero-E6 cells. Data are means ± SEM of triplicate samples from a representative experiment. (D and E) Representative ELISpot images showing IFN-γ production by (D) splenocytes and (E) pneumonocytes stimulated with 400 μg/mL S1 or S2 protein.


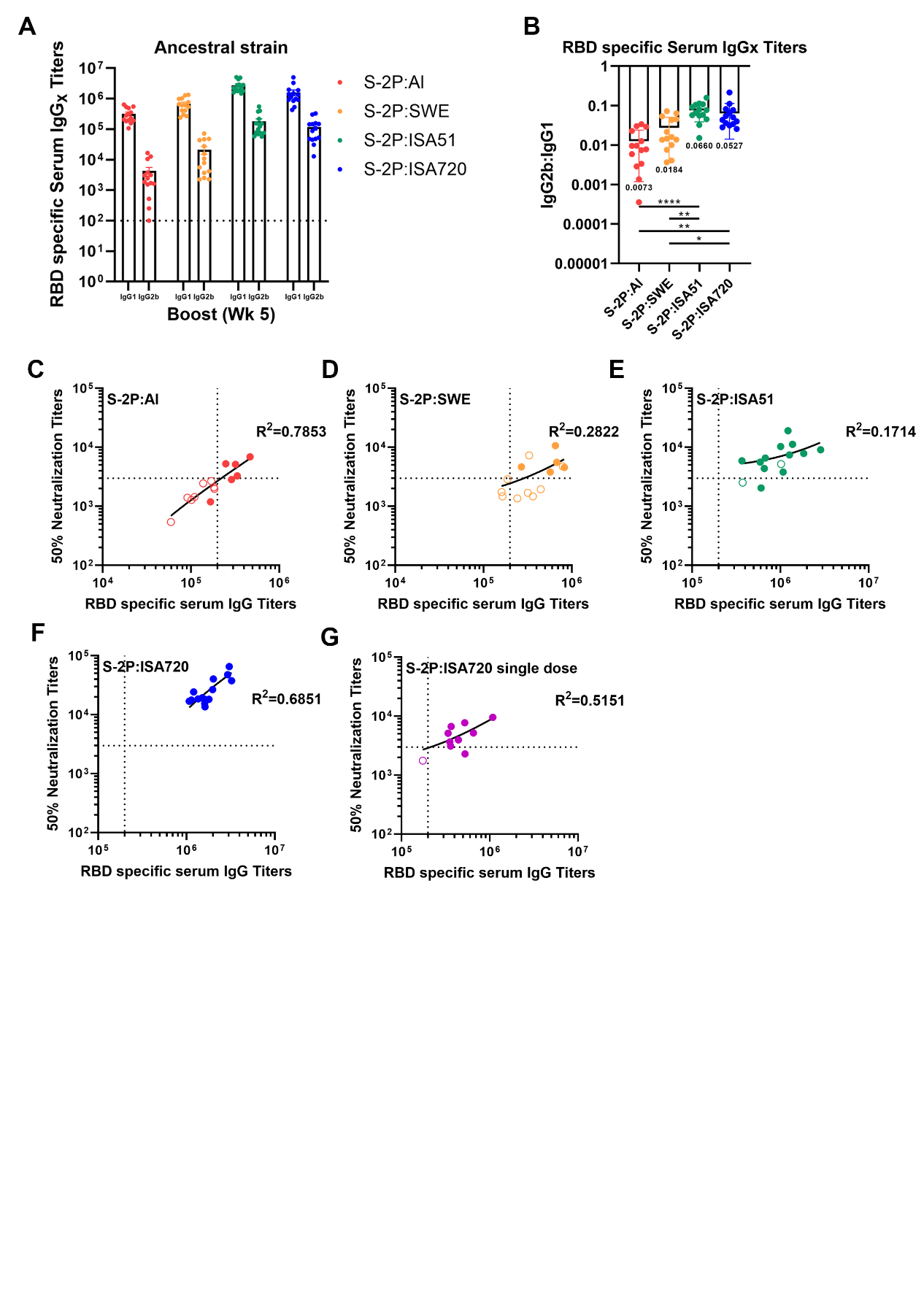


**Fig S2** Anti-RBD IgG subclass, anti-RBD IgG and neutralizing antibody assay of immunized hACE2 mice. (A) Anti-RBD IgG1 and IgG2b antibody titers at week 5 post-prime immunization. Fourteen hACE2 mice per group were vaccinated at weeks 0 and 3 with different vaccine formulations. Serum antibody levels were measured by ELISA (n=14). (B) IgG2b:IgG1 ratio for each mouse across groups. (C-G) The correlation analysis of serum anti-RBD IgG titers and corresponding neutralizing antibody titers. Data shown as mean ± SEM. Groups were compared using one-way ANOVA with Tukey’s multiple comparisons test.


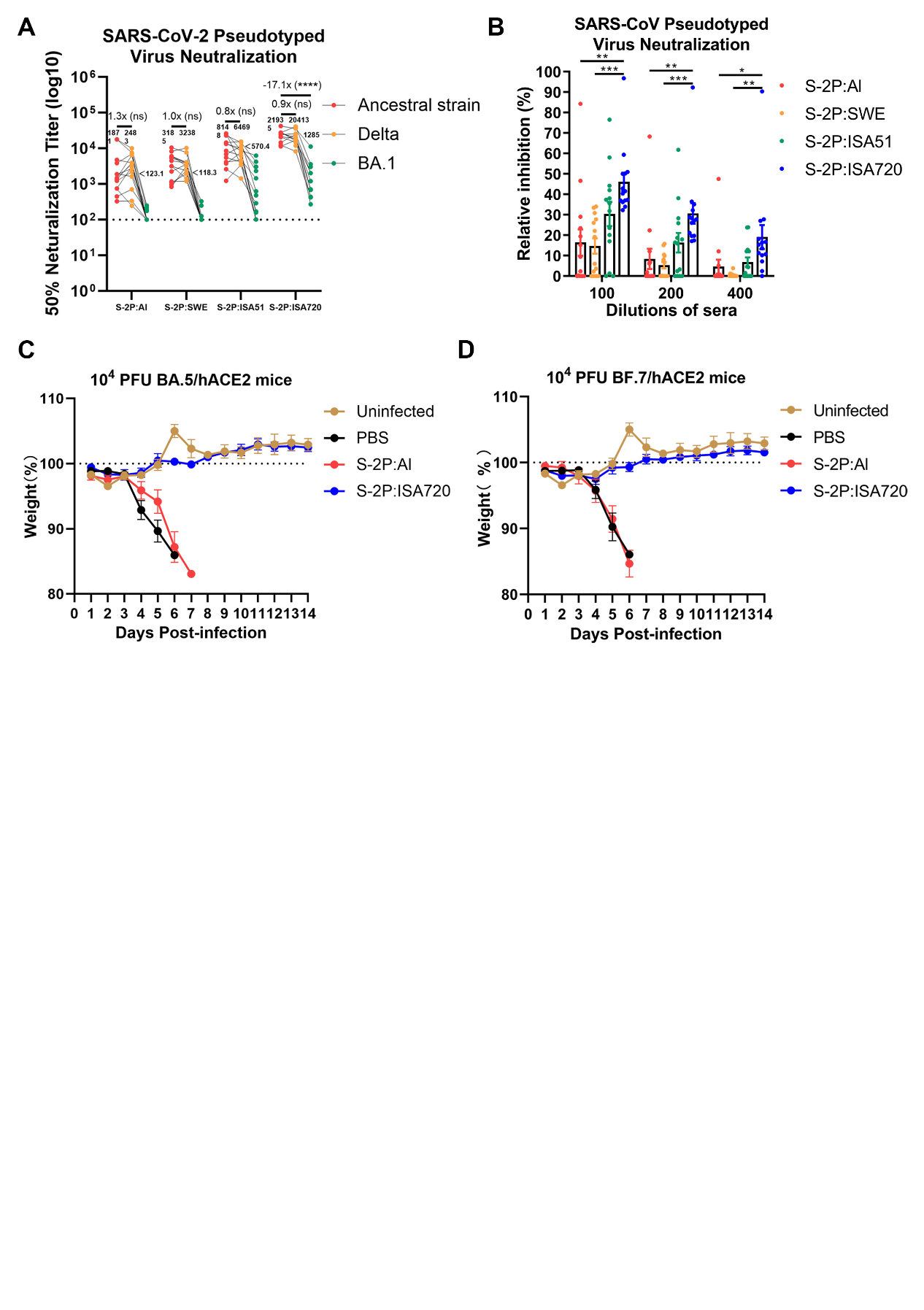


**Fig S3** Adjuvant effects on serum cross-neutralization. (A) Cross-neutralizing activity in sera from hACE2 mice immunized with different adjuvanted S-2P formulations (n=11–14/group) was evaluated at week 5 post-prime using pseudotyped virus assays; sample sizes varied between assays due to insufficient serum volume for all tests. (B) SARS-CoV neutralization at indicated serum dilutions (1:100, 1:200, 1:400). (C and D) Body weight changes post-infection with Omicron BA.5 (C) or BF.7 (D). Data are represented as the mean ± SEM. Groups were compared using one-way ANOVA with Tukey’s multiple comparisons test.


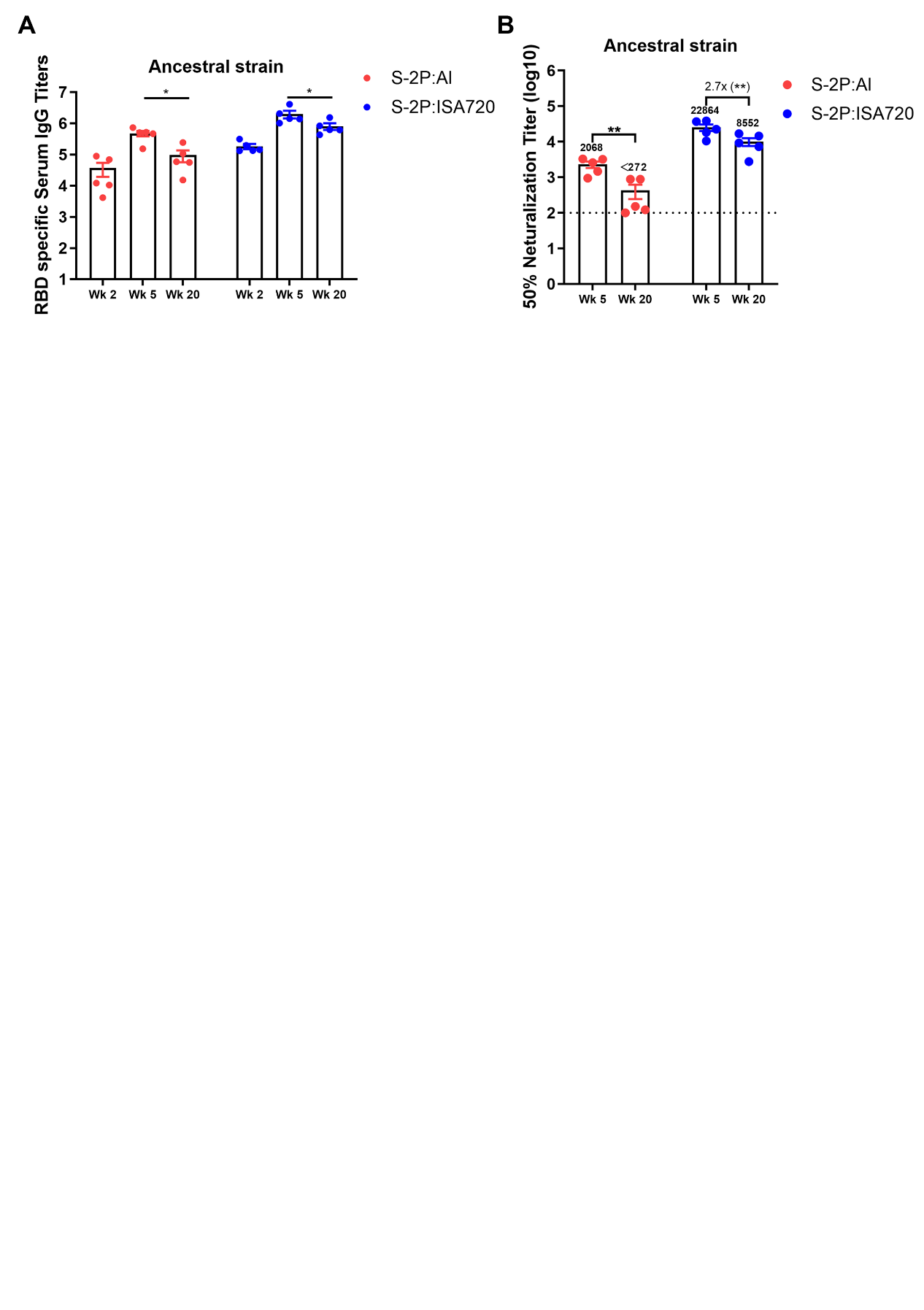


**Fig S4** (A) Anti-RBD IgG and (B) neutralizing antibody titers at weeks 5 and 20 of hACE2 mice received prime-boost immunization with 3-week interval. Groups were compared using one-way ANOVA with Tukey’s multiple comparisons test or unpaired two-sided Student’s t-test.


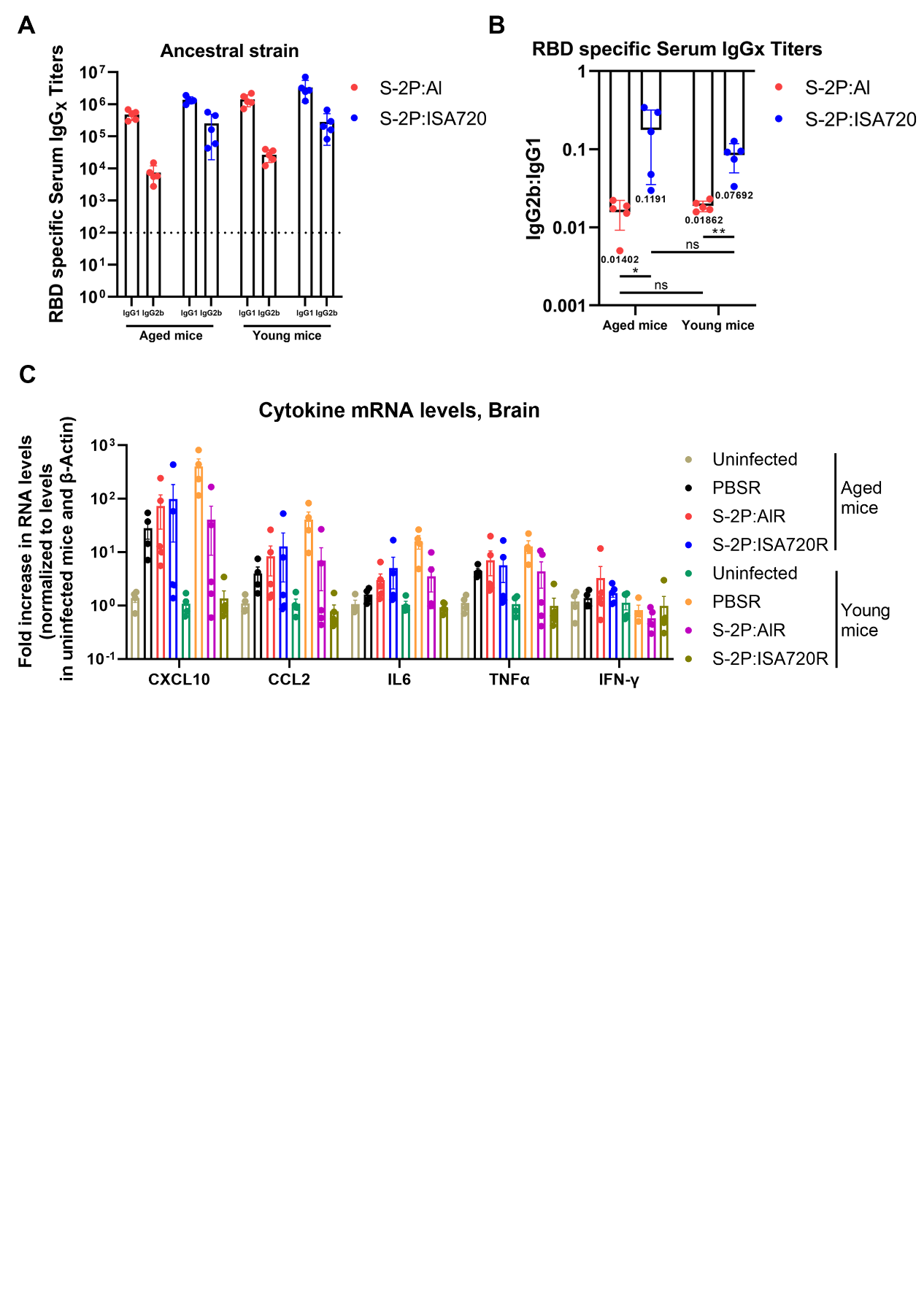


**Fig S5** Anti-RBD IgG subclass and brain inflammatory factors mRNA expression of aged and young mice. (A) Anti-RBD IgG1 and IgG2b antibody titers at week 5 post-immunization. 3- and 18-month-old hACE2 mice (n=5 per group) were prime/boost-vaccinated with S-2P:Al or S-2P:ISA720 at weeks 0 and 3. Serum antibody levels were measured by ELISA. Data points represent individual mice. (B) IgG2b:IgG1 ratio distribution across experimental groups. (C) The relative mRNA levels of neuroinflammatory cytokines in mice 4 days after viral challenge. Statistical analysis was performed using one-way ANOVA with Tukey's multiple comparisons test.
